# The 3′ processing of antisense RNAs physically links to chromatin-based transcriptional control

**DOI:** 10.1073/pnas.2007268117

**Published:** 2020-06-15

**Authors:** Xiaofeng Fang, Zhe Wu, Oleg Raitskin, Kimberly Webb, Philipp Voigt, Tiancong Lu, Martin Howard, Caroline Dean

**Affiliations:** ^a^Department of Cell and Developmental Biology, John Innes Centre, NR4 7UH Norwich, United Kingdom;; ^b^Wellcome Centre for Cell Biology, School of Biological Sciences, University of Edinburgh, EH9 3BF Edinburgh, United Kingdom;; ^c^Computational and Systems Biology, John Innes Centre, NR4 7UH Norwich, United Kingdom

**Keywords:** non-coding RNA, chromatin, polycomb, *FLC*, *Arabidopsis*

## Abstract

Noncoding RNA plays essential roles in transcriptional control and chromatin silencing. At *Arabidopsis thaliana FLC,* antisense transcription quantitatively influences transcriptional output, but the mechanism by which this occurs is still unclear. Proximal polyadenylation of the antisense transcripts by FCA, an RNA-binding protein that physically interacts with RNA 3′ processing factors, reduces *FLC* transcription. This process genetically requires FLD, a homolog of the H3K4 demethylase LSD1. However, the mechanism linking RNA processing to FLD function had not been established. Here, we show that FLD tightly associates with LUMINIDEPENDENS (LD) and SET DOMAIN GROUP 26 (SDG26) in vivo, and, together, they prevent accumulation of monomethylated H3K4 (H3K4me1) over the *FLC* gene body. SDG26 interacts with the RNA 3′ processing factor FY (WDR33), thus linking activities for proximal polyadenylation of the antisense transcripts to FLD/LD/SDG26-associated H3K4 demethylation. We propose this demethylation antagonizes an active transcription module, thus reducing H3K36me3 accumulation and increasing H3K27me3. Consistent with this view, we show that Polycomb Repressive Complex 2 (PRC2) silencing is genetically required by FCA to repress *FLC*. Overall, our work provides insights into RNA-mediated chromatin silencing.

## Significance

RNA-mediated chromatin regulation is central to gene expression in many organisms. However, the mechanisms by which RNA influences the local chromatin environment are still poorly understood. Here, we show how RNA 3′ processing factors, which promote proximal polyadenylation of an *Arabidopsis* antisense transcript, physically associate with the chromatin modifiers FLD/LD/SDG26. The chromatin modifiers exist in a protein complex that inhibits H3K4me1 and H3K36me3 accumulation. By antagonizing transcription, the FLD/LD/SDG26-containing complex promotes H3K27me3 accumulation, reducing transcriptional initiation and elongation rates. This cotranscriptionally mediated chromatin silencing mechanism may be widely relevant for gene regulation in many organisms.

Both long and short noncoding chromatin-associated RNA transcripts have emerged as key regulators of the chromatin environment (1). Detailed mechanisms of how 21- to 24-nt RNAs initiate and maintain heterochromatin have been elucidated (2). However, less is understood about the mechanisms linking long noncoding RNA, chromatin regulation, and transcription. The most well-studied example is the role of X inactive specific transcript (*Xist*) in X chromosome inactivation (3). Different repeats on *Xist* recruit an array of protein factors that silence and conformationally alter the X chromosome (4). The RNA-binding protein SPEN binds the *Xist* A repeat and has recently been shown to transcriptionally down-regulate X-linked genes and trigger Polycomb silencing in a process requiring nucleosome remodelers and histone deacetylases (5). Similar RNA-mediated chromatin mechanisms act at the single locus *Arabidopsis FLOWERING LOCUS C* (*FLC*), which encodes a MADS-box transcription factor that acts as a floral repressor in *Arabidopsis thaliana*. A well-understood process involving *FLC* is vernalization, the cold-induced epigenetic silencing that occurs during winter, enabling plants to flower in spring. Cold induces a set of antisense long noncoding transcripts at the *FLC* locus, called *COOLAIR*, which mediate transcriptional down-regulation of *FLC*, as a prelude to a Polycomb-induced epigenetic switch (6). However, in a second less well understood mechanism at *FLC*, transcription is quantitatively regulated by *COOLAIR* antisense transcript processing linked to chromatin regulation. This is mediated by a set of genes grouped into the autonomous floral pathway (some of which are putative equivalents of SPEN), which have widespread transcriptional functions in the *Arabidopsis* genome through RNA-mediated chromatin pathways (7).

The autonomous pathway component FCA is an RNA-binding protein that mediates alternative 3′ end processing of *COOLAIR* transcripts (8). FCA associates with a coiled-coil protein, FLL2, which promotes formation of liquid-like nuclear condensates that appear to concentrate 3′ processing factors and change their dynamics at specific poly(A) sites (9). The proximal processing of *COOLAIR* results in an *FLC* chromatin environment that reduces *FLC* transcriptional initiation and elongation rates (10). This process requires FLOWERING LOCUS D (FLD), which is a homolog of the H3K4 demethylase LSD1 (11). Nevertheless, how FCA-mediated RNA processing links to FLD remained to be elucidated.

We have investigated this mechanism further, and here we identify two proteins, LUMINIDEPENDENS (LD) and SET DOMAIN GROUP 26 (SDG26), that tightly associate with FLD. Like FLD, LD and SDG26 function genetically in the *FLC*-repression pathway with FCA. We find that SDG26 transiently interacts with FY, one of the RNA 3′ processing factors that associates with FCA, physically linking FCA to FLD. Through genetic and chromatin immunoprecipitation analysis, we determine that loss of the FLD/LD/SDG26, or FCA, leads to overaccumulation of histone modifications, including H3K4me1/me2 and H3K36me3. Thus, we can now physically link RNA 3′ processing of the *COOLAIR* transcripts with a chromatin modification complex that influences H3K4me1-H3K36me3 and transcriptional activity at the locus. By antagonizing transcription, FLD/LD/SDG26-containing complex promotes H3K27me3 accumulation, consistent with a requirement for Polycomb Repressive Complex 2 in the FCA-mediated repression of *FLC*. We propose that FLD/LD/SDG26 influences an active transcription module that antagonizes PRC2 function.

## Results

### FLD Associates with LD and SDG26.

**Fig. 1.**
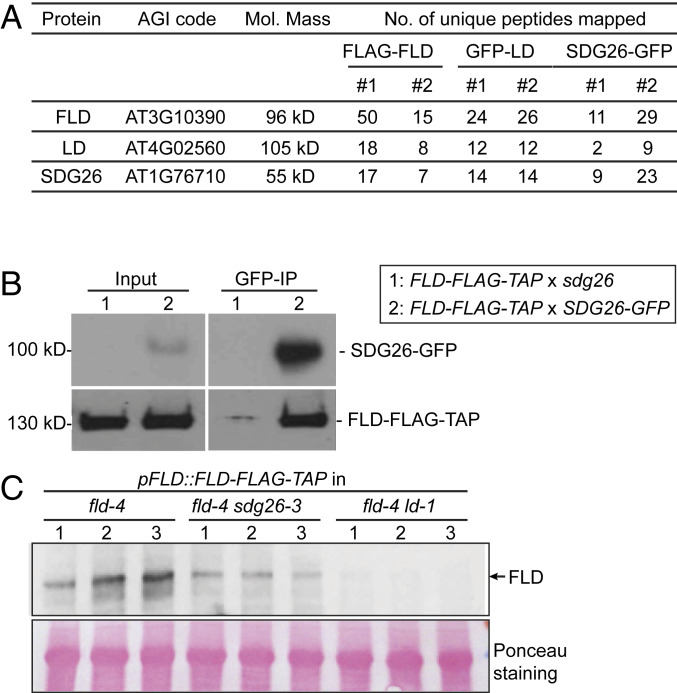
FLD forms a complex with LD and SDG26. (*A*) Table listing the number of unique peptides identified for FLD, LD, and SDG26 in FLAG-FLD, GFP-LD, and SDG26-GFP affinity purifications. Nontransgenic Col-0 was included in all purifications, and the transgenic line expressing GFP alone was included in GFP purifications as a negative control. The read counts from the negative controls were all zero for the listed proteins. (*B*) Co-IP in stable transgenic plants to detect the association of SDG26-GFP with FLD-FLAG-TAP. The *FLD-FLAG-TAP* transgenic line was crossed either with *sdg26* mutant or *SDG26-GFP* transgenic line. F1-generation plants were used for co-IP. (*C*) The protein level of FLD-FLAG-TAP in the indicated genetic backgrounds as determined by Western blot. The numbers indicate three biological replicates. Ponceau staining served as a loading control.

We previously performed a suppressor mutagenesis screen and identified FLD as one of the components required for FCA-mediated *FLC* regulation (11). To gain insights into how FLD represses *FLC* transcription, we used a proteomic approach to search for FLD interactors. We immunopurified FLD from a transgenic line expressing FLD tagged at the carboxyl terminus with FLAG-TAP epitopes (FLD-FLAG-TAP) (10). Mass spectrometric analyses of the FLD immunoprecipitation revealed that FLD tightly associates with LUMINIDEPENDENS (LD) and a SET domain protein, SDG26, in vivo (Fig. 1*A* and Dataset S1). Purifications from transgenic plants expressing GFP-tagged versions of each protein but not GFP only or Col-0 enriched the other two proteins of the complex (Fig. 1*A* and Datasets S2 and S3). The interaction between FLD and SDG26 was confirmed by coimmunoprecipitation (co-IP) in stable transgenic lines (Fig. 1*B*). Loss of LD or SDG26 caused a reduction in FLD protein levels (Fig. 1*C* and *SI Appendix*, Fig. S1). One possible explanation for this is that the interaction between FLD and LD/SDG26 may be required for FLD stability.

LD was one of the first flowering regulators to be cloned based on a late-flowering phenotype of a T-DNA insertion (12), but how its function connected to other autonomous pathway components was unclear. LD encodes a protein carrying an N-terminal homeodomain (*SI Appendix*, Fig. S2*A*) and has been reported to bind DNA without sequence specificity (13). SDG26 is a close homolog of SDG8 (*SI Appendix*, Fig. S2*A*), the major histone H3K36 methyltransferase in the *Arabidopsis* genome; however, in vitro and in vivo analysis so far has provided no evidence that SDG26 is an H3K36 methyltransferase. In fact, *sdg26* mutants show an opposite (late-flowering) phenotype compared to *sdg8* (early flowering) through opposite effects on *FLC* expression, suggesting different functions or indirect effects (14, 15). We tested the subcellular localization of FLD, LD, and SDG26 in stable transgenic lines and found that they are all nuclear-localized (*SI Appendix*, Fig. S2*B*).

### LD and SDG26 Function Genetically in the Same Pathway as FLD and FCA.

**Fig. 2.**
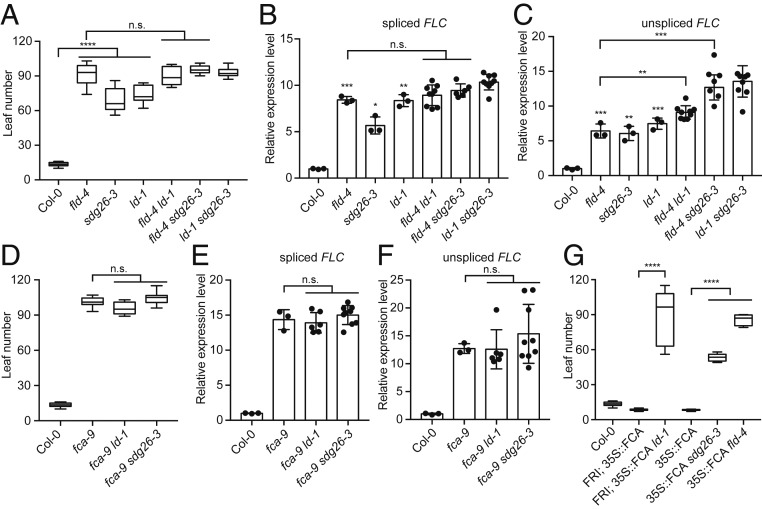
The FLD/LD/SDG26 complex functions genetically downstream of FCA to repress *FLC*. (*A*) Flowering time of indicated plants (assayed as total leaf number, produced by the apical meristem before it switched to producing flowers) grown in a long-day photoperiod. Data are presented as the mean ± SD (*n* ≥ 10). Asterisks indicate significant differences between the indicated plants (****P ≤ 2.42353E-09, two-tailed *t* test). n.s., not significant. (*B* and *C*) Expression of spliced *FLC* (*B*) and unspliced *FLC* (*C*) relative to wild-type Col-0 in the indicated mutants. Data are presented as the mean ± SD (*n* ≥ 3). Asterisks indicate significant differences between the indicated plants (*P ≤ 0.0121, **P ≤ 0.0028, ***P ≤ 0.0010, two-tailed *t* test). n.s., not significant. Each dot represents one biological replicate. (*D*) Flowering time of indicated plants grown in a long-day photoperiod. Data are presented as the mean ± SD (*n* ≥ 10). n.s., not significant. (*E* and *F*) Expression of spliced *FLC* (*E*) and unspliced *FLC* (*F*) relative to wild-type Col-0 in the indicated mutants. Data are presented as the mean ± SD (*n* ≥ 3). n.s., not significant. Each dot represents one biological replicate. (*G*) Flowering time of indicated plants grown in a long-day photoperiod. Data are presented as the mean ± SD (*n* ≥ 10). Asterisks indicate significant differences between the indicated plants (****P ≤ 4.68856E-14, two-tailed *t* test).

Similar to *fld* mutant, loss-of-function mutations of *LD* and *SDG26* showed a late-flowering phenotype and increased *FLC* expression (Fig. 2 *A*–*C*). In order to dissect the genetic relationships between FLD, LD, and SDG26, we combined the mutations to create double mutants. The results showed that *fld ld*, *fld sdg26*, and *ld sdg26* did not give any additional lateness (Fig. 2*A*) or increase in spliced *FLC* RNA levels (Fig. 2*B*), but did lead to higher unspliced *FLC* RNA levels (Fig. 2*C*), compared to the single mutants. The inconsistency between spliced and unspliced *FLC* suggests that, similar to Paf1C (16), FLD, LD, and SDG26 might have a concerted role in regulating the release of nascent *FLC* transcripts.

FLD has been shown to function in the same genetic pathway and downstream of FCA in that *fld* is not additive to *fca* with respect to flowering time, and *fld* suppressed the ability of FCA to down-regulate *FLC* (11). To test whether LD and SDG26 behave in the same way as FLD, we first combined *ld* and *sdg26* with *fca* and found no additivity compared to *fca* with respect to flowering time (Fig. 2*D*) or *FLC* expression (Fig. 2 *E* and *F*). Combination of a *35S-FCA* transgene, with and without the *FLC* activator FRIGIDA, with *ld* and *sdg26* mutations then showed that both mutations compromised the effect of overexpressed FCA on *FLC* (Fig. 2*G*). Taken together, these data support that FLD, LD, and SDG26 exist in a complex that functions downstream of FCA to repress *FLC* expression.

### SDG26 Transiently Interacts with the 3′ Processing Factor FY (WDR33).

**Fig. 3.**
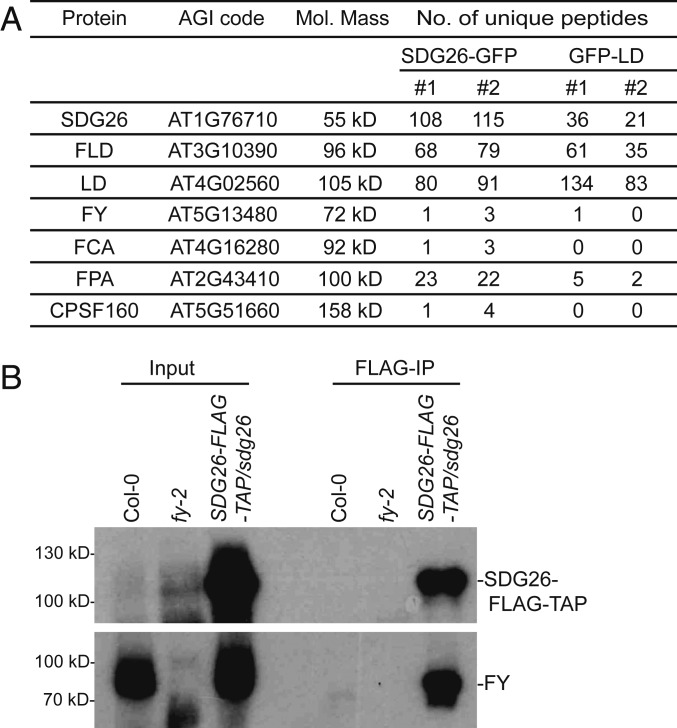
SDG26 transiently associates with 3′ processing factors. (*A*) A partial list of proteins identified by SDG26 and LD affinity purifications after cross-linking. Nontransgenic Col-0 and transgenic line expressing GFP alone were included as negative controls in both purifications, and the read counts were all zero for the listed proteins. (*B*) Co-IP in stable transgenic plants after cross-linking to detect the association of SDG26 with FY.

The strong genetic interactions between FLD/LD/SDG26 and FCA raised the question of how FCA function is molecularly linked to FLD. No in vivo physical interactions of FCA with 3′ processing factors or chromatin regulators had been found until our recent analysis using a technique termed cross-linked nuclear immunoprecipitation and mass spectrometry (CLNIP–MS) (9). We found FCA interacted with both RNA and a range of proteins and, in vivo, localizes to nuclear condensates that are highly dynamic (9). Those condensates are likely to concentrate 3′ processing factors and contribute to 3′-end processing of RNAs including *COOLAIR* (9). We reasoned that the interaction between the FLD/LD/SDG26-containing complex and FCA, if any, would also be transient and dynamic. To this end, we applied CLNIP–MS to SDG26. Surprisingly, we found that, in addition to finding FLD and LD with high peptide counts, some 3′ RNA processing factors were also detected (Fig. 3*A* and Dataset S4) in the SDG26 immunoprecipitation after cross-linking. These include FCA, as well as the RRM-containing protein FPA (8, 17), FY (18, 19), and Cleavage/Polyadenylation Specificity Factor 160 (CPSF160), all of which have been shown to associate with FCA and colocalize with FCA in the nuclear condensates (9). Purifications from Col-0 or a transgenic plant expressing a 35S-GFP fusion did not retrieve any of those proteins (Dataset S4). We then set out to confirm the interaction between SDG26 and FY, using an FY antibody raised in rabbits against the native recombinant protein (20). Using an *SDG26-FLAG-TAP* transgenic line, we performed cross-linked nuclear immunoprecipitation of SDG26 and probed against FY. The result showed that FY was readily detected (Fig. 3*B*). Without cross-linking, neither FY nor any of the 3′ processing factors were found in the SDG26 immunoprecipitation (Dataset S3). CLNIP-MS of LD also identified FY and FPA (Fig. 3*A* and Dataset S5). These data suggest that the interactions between the FLD/LD/SDG26-containing complex and 3′ processing factors provide a physical link, so that, when 3′ RNA processing of proximal *COOLAIR* occurs, the FLD/LD/SDG26-containing complex is brought in to repress *FLC* transcription.

### Loss of FLD/LD/SDG26 Results in Overaccumulation of H3K4me1 at *FLC*.

**Fig. 4.**
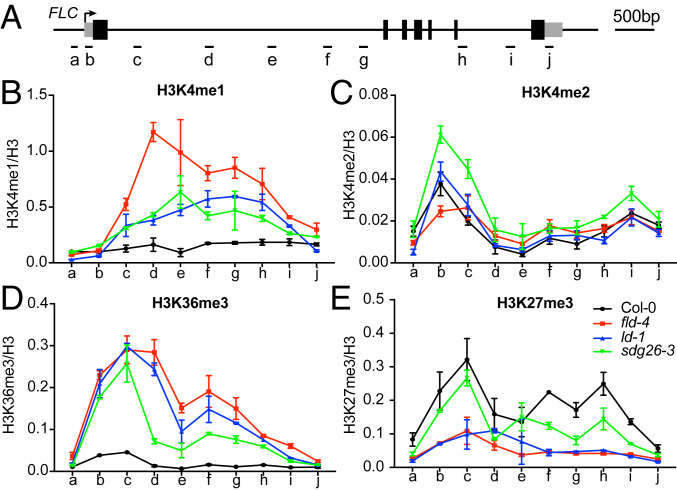
Measurements of histone modification levels upon the loss of the FLD/LD/SDG26 complex. (*A*) Schematic diagram showing *FLC* gene structure. Gray boxes represent untranslated regions, and black boxes represent exons. The other regions are represented by the black line. The arrow indicates the transcription start site (TSS). Short black lines indicate positions of primers used for qPCR amplification. (*B*–*E*) ChIP analysis of H3K4me1 (*B*), H3K4me2 (*C*), H3K36me3 (*D*), and H3K27me3 (*E*) levels at *FLC* in various genetic backgrounds. The letters on the *x* axis correspond to the positions indicated in *A*. Data are shown as relative to H3. Values are means ± SEM from three independent biological replicates.

Our mathematical modeling and experimental evidence have shown that FLD-mediated repression of *FLC* is achieved in a manner consistent with a coordinated reduction of transcription initiation and Pol II elongation rates (10). Whether and how this is connected to histone modifications is not fully understood. *Arabidopsis* has four homologs of human LSD1, including FLD, LDL1, LDL2, and LDL3 (21). The *fld* mutation led to a limited 1.5- to 2-fold increase of H3K4me2 on *FLC* (10, 11). More recently, the *ldl2* mutation was shown to increase gene body H3K4me1, which correlated positively with gene expression (22). We therefore decided to analyze the effect of *FLD*, *LD*, and *SDG26* mutations on H3K4me1 and H3K4me2 levels at *FLC*. Chromatin immunoprecipitation coupled with quantitative PCR (ChIP-qPCR) showed a small increase of H3K4me2 at 1 to 4 kb beyond the TSS of *FLC* in *fld* (Fig. 4 *A* and *C*), consistent with previous reports (10, 11). Surprisingly, we observed a much more dramatic increase of H3K4me1 over the *FLC* gene body in *fld* (Fig. 4 *A* and *B*). *ld* and *sdg26* also significantly overaccumulated H3K4me1 (Fig. 4*B*), indicating a major role of the FLD/LD/SDG26-containing complex in inhibiting H3K4me1 accumulation through the demethylase activity of FLD. It is also noteworthy that *sdg26* accumulated more H3K4me2 than *fld* (Fig. 4*C*), suggesting a role for the FLD/LD/SDG26-containing complex in a stepwise removal of H3K4me2 and H3K4me1, with each component contributing differently to this activity. *fca-9* showed a large increase in H3K4me1 and a similar increase in H3K4me2 as *sdg26*, in agreement with FLD/LD/SDG26 functioning genetically downstream of FCA (*SI Appendix*, Fig. S3 *A*–*C*). Given that SDG26 features a SET domain, a hallmark of histone methyltransferases, we sought to determine whether the FLD/LD/SDG26-containing complex, in addition to FLD-mediated demethylation, could also directly alter chromatin states through SDG26-mediated histone methylation. However, we failed to detect activity of SDG26 toward recombinant *Arabidopsis* nucleosomes in vitro for both heterologously expressed SDG26 or FLD/LD/SDG26 complex purified from Sf9 cells, nor for the endogenous FLD/LD/SDG26-containing complex purified via FLD-FLAG-TAP purification (*SI Appendix*, Fig. S4). Overall, these findings suggest demethylation of H3K4 is a major activity of the complex.

### SDG8 Is Epistatic to FLD/LD/SDG26 to Activate *FLC*.

**Fig. 5.**
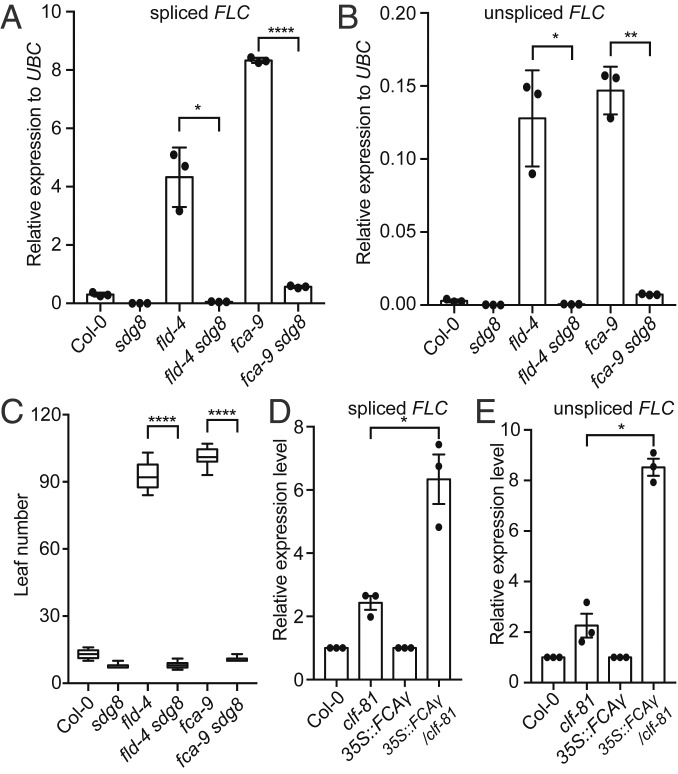
The genetic relationships of *FCA* and *FLD* with *SDG8* and PRC2. (*A* and *B*) Expression of spliced *FLC* (*A*) and unspliced *FLC* (*B*) relative to *UBC* in the indicated genotypes. Data are presented as the mean ± SD (*n* = 3). Asterisks indicate significant differences between the indicated plants (*P ≤ 0.0217, **P = 0.0043, ****P = 6.27105E-05, two-tailed *t* test). Each dot represents one biological replicate. (*C*) Flowering time of indicated plants (assayed as total leaf number, produced by the apical meristem before it switched to producing flowers) grown in a long-day photoperiod. Data are presented as the mean ± SD (*n* ≥ 10). Asterisks indicate significant differences between the indicated plants (****P ≤ 2.26769E-09, two-tailed *t* test). (*D* and *E*) Expression of spliced *FLC* (*D*) and unspliced *FLC* (*E*) relative to *UBC* in the indicated genotypes. Note that expression level in the mutant background was separately normalized to its corresponding wild-type background. Data are presented as the mean ± SD (*n* = 3). Asterisks indicate significant differences between the indicated plants (*P ≤ 0.0458, two-tailed *t* test). Each dot represents one biological replicate.

H3K4me1 is enriched at enhancers as well as gene bodies in mammalian cells (23). Recent studies suggested that H3K4me1 might fine-tune, rather than tightly control, enhancer activity and function (24–26). In plants, H3K4me1 is mainly found in gene bodies, removal of which mediates transcriptional silencing (22). Interestingly, the CW domain of *Arabidopsis* SDG8, an H3K36me3 methyltransferase, preferentially binds H3K4me1 (27, 28), providing a mechanism to link H3K4me1 to delivery of the active histone modification H3K36me3. Consistent with this, we found loss of the FLD/LD/SDG26-containing complex, as well as FCA, led to a large overaccumulation of H3K36me3 in the *FLC* gene body (Fig. 4*D* and *SI Appendix*, Fig. S3*D*), which mirrored the change of H3K4me1 (Fig. 4*B* and *SI Appendix*, Fig. S3*B*). In addition, H3K27me3, the mutually exclusive histone modification of H3K36me3, was greatly reduced in the *fld-4*, *ld*, and *fca* mutants (Fig. 4*E* and *SI Appendix*, Fig. S3*E*). Consistent with this, SDG8 ChIP did not show signal on *FLC* in the Col-0 background (29), where H3K4me1 was kept at a very low level (Fig. 4*B*). The connection between H3K4me1 and H3K36me3 raised the possibility that FLD/LD/SDG26 repressed *FLC* via removal of H3K4 methylation, thereby inhibiting SDG8-mediated H3K36me3 and indirectly promoting the accumulation of H3K27me3. To test this possibility, we generated the *fld sdg8* double mutant and found that the *sdg8* mutation completely suppressed both the *fld*-induced higher expression of *FLC* (Fig. 5 *A* and *B*) and the resulting delayed flowering time (Fig. 5*C*). This would suggest that the FLD/LD/SDG26 repression of *FLC* transcription involves inhibition of SDG8 function. In comparison, the *sdg8* mutation largely, but not completely, reversed the expression of *FLC* (Fig. 5 *A* and *B*) and flowering time (Fig. 5*C*) caused by *fca-9*, suggesting that FCA can, to a limited extent, also repress *FLC* via a pathway that is independent of FLD and SDG8.

### FCA Requires PRC2 to Silence *FLC*.

The above data support a model where the alternative 3′ processing of *COOLAIR* by FCA mediates the silencing of *FLC* by Polycomb Repressive Complex 2 (PRC2) via inhibiting an active transcription module consisting of H3K4me1, H3K36me3, and transcription, which antagonizes the deposition of H3K27me3 (30). We tested this model by asking whether PRC2 is required by FCA to silence *FLC*. We took advantage of an *Arabidopsis* progenitor line carrying a single insertion of a *35S*::*FCAγ* transgene in combination with an active *FRIGIDA* allele, in an otherwise wild-type background, which we had used to identify mutations suppressing the ability of FCA to down-regulate *FLC* (11). This sensitized background enhances *FLC* derepression and so is an efficient way to screen for factors required for FCA function. A weak allele of *clf*, reduced in PRC2 H3K27me3 methyltransferase activity (31), was introduced into this *35S*::*FCAγ* genotype. *clf-81* strongly released *FLC* expression, much more than in the Col background (Fig. 5 *D* and *E*), supporting that FCA requires PRC2 to silence *FLC*. In line with our findings, Tian et al. showed that CLF enrichment at the *FLC* locus requires FCA function (32).

## Discussion

Studying the quantitative transcriptional regulation of the *A. thaliana* floral repressor *FLC* has led us into dissection of how alternative processing of antisense transcripts regulates local chromatin environment and thus transcriptional output (7). We find that dynamic interactions between RNA-binding proteins, 3′ processing factors, and the chromatin modifiers FLD/LD/SDG26 result in a chromatin state associated with low transcriptional initiation and slow elongation, marked by low H3K4me1, low H3K36me3, and high H3K27me3. Loss of any of the factors switches the locus to the opposing high transcriptional state, overaccumulation of H3K4me1 and H3K36me3 and reduction of H3K27me3. We propose that the FLD/LD/SDG26 exist in a complex that inhibits an active transcription module, so promoting the deposition of H3K27me3 (*SI Appendix*, Fig. S5). This process parallels with the cleavage and polyadenylation factor (CPF)-mediated facultative heterochromatin assembly in yeast (33), the exact mechanism of which is still unknown.

FCA associates dynamically with 3′ processing factors in FCA nuclear bodies (9). The fact that the interactions between SDG26 and 3′ processing factors were only detected after cross-linking suggested that the interactions are also dynamic, and raised the possibility that FLD/LD/SDG26 might colocalize in FCA nuclear bodies. LD, like FCA and FY, has been found to contain a prion-like domain (34) (*SI Appendix*, Fig. S2*A*), which was identified as a driver for ribonucleoprotein granule assembly (35), and LD formed distinct foci when expressed in yeast cells (34). However, under normal confocal microscopy and expressed at endogenous levels, neither FLD, LD, nor SDG26 formed obvious nuclear bodies (*SI Appendix*, Fig. S2*B*). One possible explanation is that FLD/LD/SDG26 form nuclear bodies in vivo that are too dynamic/small to be detected by normal confocal microscopy. Superresolution microscopy analysis of FLD, LD, and SDG26 subcellular localization will help to address this question. On the contrary, not all genes in the genome targeted by FCA for RNA processing also need the FLD/LD/SDG26-containing complex for silencing (36). This agrees with our finding that FCA immunoprecipitation after cross-linking did not recover FLD, LD, or SDG26 (9). In addition, genetic data suggested that, even at the *FLC* locus, FCA could function in FLD-independent pathways to achieve some measure of silencing (Fig. 5 *A* and *B*) (11). A recent study showed that FCA interacts with CLF in vitro and in vivo, suggesting an FLD-independent role of FCA in regulating H3K27me3 directly (32). However, we have not detected this interaction in FCA on in vivo immunoprecipitation-mass spectrometry (IP-MS) (9), and it was not detected in CLF on in vivo IP-MS (37).

An important question raised by this work is what is the active transcription module that FLD/LD/SDG26-containing complex inhibits. We were unable to find any histone methyltransferase activity in vitro for the FLD/LD/SDG26 complex (*SI Appendix*, Fig. S4), suggesting that additional components are required for the complex to exert its function. One tantalizing hypothesis is that the histone-modifying activity is tightly linked to the RNA polymerase II (Pol II) complex during transcription. Indeed, we detected Pol II subunits (e.g., NRPB1, NRPB2, and NRPB3) and factors involved in the regulation of transcription initiation and elongation (e.g., SPT5, SPT6, and SPT16) in the SDG26 CLNIP-MS list (Dataset S4). In addition, LD contains a PP1-AP–like domain shared with the transcription elongation factor TFIIS, suggesting a role for LD in transcriptional elongation (38). Further analysis of these possibilities will expand our understanding of how the RNA-binding protein FCA connects *COOLAIR* to antagonizing an active transcription module, thereby eventually leading to Polycomb silencing. Full dissection of this mechanism will reveal any further parallels between *COOLAIR* and *Xist* function, thus elaborating our evolutionary understanding of RNA-mediated chromatin silencing.

## Materials and Methods

More detailed descriptions of the materials and methods used in this study are provided in the *SI Appendix*. A brief summary is provided here.

### Plant Materials.

The progenitor lines C2 and *35S*::*FCA*/Col (11) and the mutants *fld-4* and *fca-9* (11), *sdg8* (39), and *clf-81* (40) were described previously. The transfer-DNA (T-DNA) insertion line *ld-1* (CS876430) and *sdg26-3* (GK-087B12) were obtained from the Nottingham *Arabidopsis* Stock Centre.

### Flowering Time Analysis.

The flowering time was determined essentially as described (9). Briefly, plants were grown in long-day conditions, and the total leaf number (TLN) produced before the initiation of flowering was counted to measure variation in flowering time.

### RNA Analysis.

RNA analysis was performed as described previously (9). Briefly, total RNA was extracted, treated with DNase, and reverse-transcribed by SuperScript IV Reverse Transcriptase (Invitrogen) using gene-specific reverse primers. Quantitative reverse transcription and PCR (qPCR) analysis was performed on a LightCycler480 II (Roche), and qPCR data were normalized to *UBC*. Primer pairs for amplifying unspliced *FLC*, spliced *FLC*, and *UBC* are listed in *SI Appendix*, Table S1.

### Immunoprecipitation and Immunoblot.

Extracts were prepared and immunoprecipitated with either anti-FLAG M2 Magnetic Beads (Sigma-Aldrich; M8823) or GFP-Trap Magnetic Agarose (ChromoTek; gtma-10).

For immunoblot analysis, protein extracts or immunoprecipitates were separated by SDS/PAGE, transferred to PVDF membranes, and detected by GFP (Roche; no. 11814460001), FLAG (Sigma-Aldrich; F3165), or FY (20) antibodies.

### Materials and Data Availability.

Full lists of mass spectrometry are provided as Datasets S1–S5. All of the other raw data and materials that support the findings of this study are available from the corresponding authors upon reasonable request.

## Supplementary Material

Supplementary File

Supplementary File

Supplementary File

Supplementary File

Supplementary File

Supplementary File
