# Supplementary material for: The 3′ processing of antisense RNAs physically links to chromatin-based transcriptional control
Source: Proc Natl Acad Sci U S A. 2020 Jun 15;117(26):15316–21. doi: 10.1073/pnas.2007268117 (PMC7334503; doi:10.1073/pnas.2007268117)
Supplement: Supplementary File [file pnas.2007268117.sapp.pdf]

PNAS
www.pnas.org

Supplementary Information for

The 3' processing of antisense RNAs physically links to chromatin-based transcriptional control

Xiaofeng Fang[a,1*], Zhe Wu[a,2*], Oleg Raitskin[a], Kimberly M. Webb[c], Philipp Voigt[c], Tiancong Lu[a,3], Martin Howard[b], and Caroline Dean[a,1]

[a]Department of Cell and Developmental Biology, John Innes Centre, Norwich Research Park, Norwich, NR4 7UH, UK

[b]Computational and Systems Biology, John Innes Centre, Norwich Research Park, Norwich NR4 7UH, UK

[c]Wellcome Centre for Cell Biology, School of Biological Sciences, University of Edinburgh, Edinburgh, EH9 3BF, UK.

[*]Joint first authors

[1]To whom correspondence should be addressed. Email: caroline.dean@jic.ac.uk; xiaofeng.fang@jic.ac.uk

[2]Present address: SUSTech-PKU Institute of Plant and Food Science, Department of Biology, Southern University of Science and Technology, Shenzhen 518055, China

[3]Present address: Beijing ProteinWorld Biotech, No.8 Waterfront South Street, Beijing 100012, China

This PDF file includes:

Supplementary text
Figures S1 to S5
Table S1
Legends for Dataset S1 to S5
SI References

www.pnas.org/cgi/doi/10.1073/pnas.2007268117

SI Materials and Methods

Plant growth conditions

Seeds were surface sterilized in 5% v/v sodium hypochlorite for 5min and rinsed three times in sterile distilled water. The seeds were then sown on standard half-strength Murashige and Skoog (MS) medium (0.22% MS, 1% sucrose, 0.5% Phytagel, Sigma, P8169) media plates and kept at 4°C in the dark for 3 days before being transferred to long photoperiod conditions (16 h light of 120 μmol m^{-2} s^{-1}/8 h dark). All RNA and protein experiments were done using 12-day-old seedlings unless specified.

DNA constructs and generation of transgenic plants

The transgenic line *FLD-FLAG-TAP/fld-4* was described previously (1). To generate the *SDG26-eGFP/sdg26* and *SDG26-FLAG-TAP/sdg26* transgenic lines, *SDG26* genomic DNA including its promoter， *eGFP* or *FLAG-TAP* sequence and 3′ untranslated region (UTR) were amplified and sequentially inserted into the pCambia1300 vector restriction digestion and T4 DNA ligation. To generate the *eGFP-LD/ld* transgenic line, the promoter of *LD*, *eGFP* sequence and the genomic region of *LD* were amplified and sequentially inserted into the pCambia1300 vector by restriction digestion and T4 DNA ligation. All the constructs described above were electroporated into *Agrobacterium tumefaciens* GV3101 for transformation of *Arabidopsis* by the floral-dip method.

Microscopy

Microscopy was performed as described previously (3). Four-day-old seedlings grown vertically on MS plates with 1% (w/v) sucrose and 0.5% (w/v) Phytagel (Sigma- Aldrich, P8169) were removed from the plates. The root tips were cut and put on a slide. Analyses of subcellular localization were performed on Zeiss LSM780 confocal microscope using a 40x/1.2 water objective and LSM 780's GaAsP spectral detector. GFP was excited at 488 nm and detected at 491-535 nm and mCherry was excited at 587 nm and detected at 590-656 nm.

Nuclei and chromatin isolation

One gram of fine powder was resuspended in 10 mL Honda buffer (0.4 M Sucrose, 2.5% Ficoll, 5% Dextran T40, 25 mM Tris-HCl, pH 7.4, 10 mM $MgCl_2$, 0.5% Triton X-100, 0.5 mM PMSF, Proteinase inhibitor cocktail, 10 mM β-mercaptoethanol, RNaseOUT), filtered through two layers of Miracloth. The flowthrough was centrifuged and washed with Honda Buffer. After washing with Honda buffer, the pellet was rinsed with 1X PBS/1 mM EDTA and resuspended in 0.25 mL prechilled glycerol buffer (20 mM Tris-HCl, pH 7.9, 75 mM NaCl, 0.5 mM EDTA, 0.85 mM DTT, 50% glycerol, 0.125 mM PMSF, Proteinase inhibitor cocktail, 10 mM β-mercaptoethanol, RNaseOUT). 50 μL was saved as nuclear fraction. Then 0.20 mL cold nuclei lysis buffer (10 mM HEPES, pH 7.6, 1 mM DTT, 7.5 mM $MgCl_2$, 0.2 mM EDTA, 0.3 M NaCl, 1 M Urea, 1% NP-40, 0.5 mM PMSF, Proteinase inhibitor cocktail, 10 mM β-mercaptoethanol, RNaseOUT) was added to resuspended pellet and gently vortexed for 2 × 2 s, incubated on ice for 2 min.The suspension was centrifuged at 14,000 rpm at 4°C for 2 min to pellet the chromatin fraction.

Immunoprecipitation and immunoblot

Two grams of 12-day-old seedlings ground in liquid nitrogen were homogenized with 4 mL extraction buffer (50 mM Tris, pH 7.5, 150 mM NaCl, 4 mM $MgCl_2$, 0.2% Nonidet P-40, 5 mM DTT, and 1 tablet/50 mL protease inhibitor cocktail). After centrifugation, the supernatant was filtered through 0.44 μm filter and incubated at 4°C with either anti-FLAG® M2 Magnetic Beads (Sigma-Aldrich; M8823) or GFP-Trap Magnetic Agarose (ChromoTek; gtma-10) for 2 h. The beads were washed with extraction buffer five times for 5 min each time. The FLAG- and GFP-bound protein complexes were boiled at 95°C for 15 min in SDS loading buffer. The protein samples were then subject to mass spectrometry or immunoblot analysis.

For immunoblot analysis, protein extracts or immunoprecipitates were mixed with SDS loading buffer and boiled for 10 min. Proteins were separated by SDS-PAGE gels, transferred to PVDF membranes,

and detected by GFP (Roche, 11814460001), FLAG (Sigma-Aldrich; F3165) or FY (2) antibodies. After the primary antibody incubation, horseradish peroxidase (HRP)-conjugated secondary antibodies: anti-Mouse IgG (GE, NA931) and anti-Rabbit IgG (GE, NA934) were used for protein detection by chemiluminescence (Thermo Scientific, 34095).

Crosslinked nuclear immunoprecipitation and mass spectrometry (CLNIP-MS)

The CLNIP-MS was performed as described previously (3). Briefly, 12-day-old seedlings were crosslinked in 1% formaldehyde. The nuclei were isolated from the crosslinked materials and resuspended in RIPA buffer (1xPBS; 1% NP-40; 0.5% sodium deoxycholate; 0.1% SDS) and sonicated. Nuclear extract was incubated with GFP-Trap Magnetic Agarose (ChromoTek; gtma-10) for 2 h and washed sequentially with Low Salt, High Salt and TE buffers. The immunoprecipitates were boiled at 95°C for 15 min to reverse crosslinking. The protein samples were gel-purified and subjected to mass spectrometry analysis by nanoLC-MS/MS on an Orbitrap Fusion™ Tribrid™ Mass Spectrometer coupled to an UltiMate® 3000 RSLCnano LC system (Thermo Scientific, Hemel Hempstead, UK). Data were searched using Mascot server (Matrixscience, London, UK) and analyzed using the MaxQuant software.

Chromatin immunoprecipitation

Nuclei were extracted using Honda buffer as described previously (Sun et al. 2013), using 1 gram of crosslinked plant material. In all histone ChIP reactions, sonication, immunoprecipitation, DNA recovery, and purification were performed as previously described (Angel et al. 2011). The antibodies used were anti-H3 (Abcam, ab1791), anti-H3K27me3 (Millipore 07-449), anti-H3K36me3 (Abcam ab9050), anti-H3K4me2 (Millipore, 07-030) and anti-H3K4me1 (Abcam ab8895). All ChIP data were quantified by qPCR with primers listed in SI Appendix,Table S1. Values were normalized first to Input and then to H3.

In vitro histone methyltransferase assays

Recombinant SDG26 containing a C-terminal FLAG-twin-Strep tag was expressed in baculovirus-infected Sf9 cells and purified via the twin-Strep tag using Strep Tactin XT beads (IBA, Göttingen, Germany) either on its own or along with His-tagged LD and FLD. Core histones (Arabidopsis H3.1, H2A, and H2B and Xenopus H4) were expressed in *E. coli* and purified from inclusion bodies as described previously (4). Assembled histone octamers were reconstituted into nucleosome arrays via salt dialysis using a plasmid containing 12 177-bp repeats of the 601-nucleosome positioning sequence. Methyltransferase assays were performed essentially as described previously for 1 h at 23 °C (4). Recombinant murine PRC2 complex purified from Sf9 cells via a FLAG tag on EZH2 was used as a positive control.

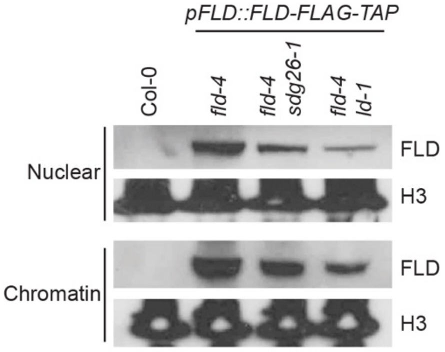

Fig. S1. Analyses of FLD protein levels by western blot. The nuclear and chromatin fractions were prepared from the indicated genotypes and analysed by western blot. Histone H3 was probed as a loading control.

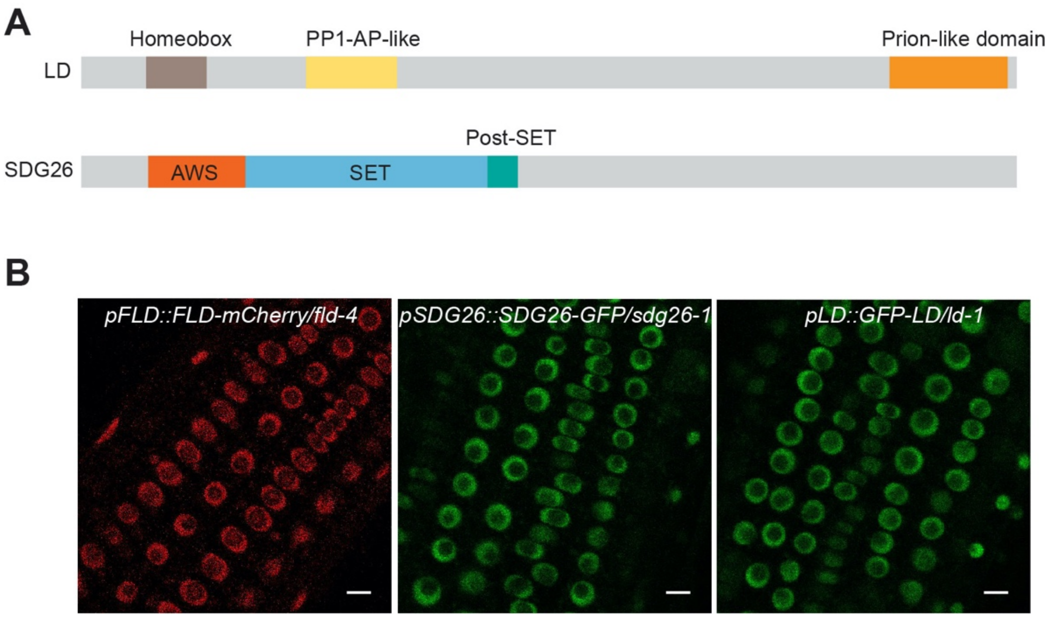

Fig. S2. Analyses of FLD, LD and SDG26. (A) The annotated functional domains of LD and SDG26. (B) The subcellular localization of FLD-mCherry, SDG26-GFP and GFP-LD in Arabidopsis root tip cells. Scale bars, 5 μm.

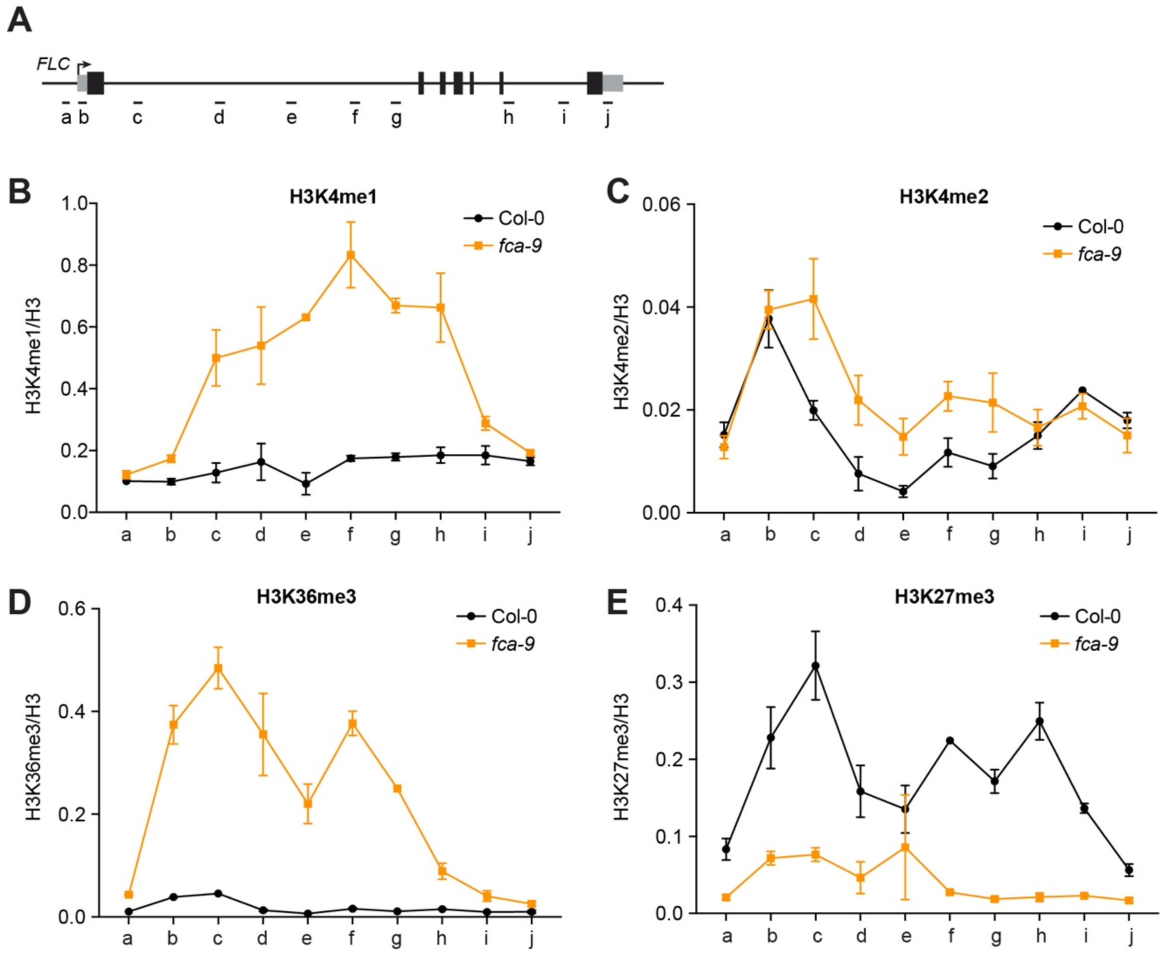

Fig. S3. The analyses of histone modifications upon the loss of the FCA. (A) Schematic diagram showing *FLC* gene structure. Gray boxes represent untranslated regions; and black boxes represent exons. The other regions are presented by the black line. The arrow indicates the transcription start site. Short black lines indicate positions of primers used for qPCR amplification. (B-E) ChIP analysis of H3K4me1 (B), H3K4me2 (C), H3K36me3 (D) and H3K27me3 (E) levels at *FLC* in various genetic backgrounds. The alphabets on the *x* axis correspond the position indicated in (A). Data are shown as relative to H3. Values are means ± SEM from three independent biological replicates.

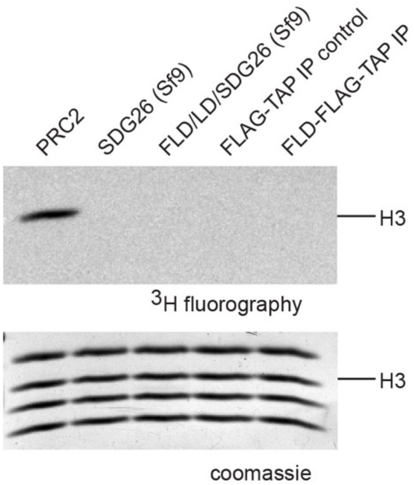

Fig. S4. No in vitro histone methyltransferase activity was detected for the FLD/LD/SDG26 complex. The indicated proteins were test towards recombinant Arabidopsis nucleosomes. Coomassie staining of histones served as a loading control.

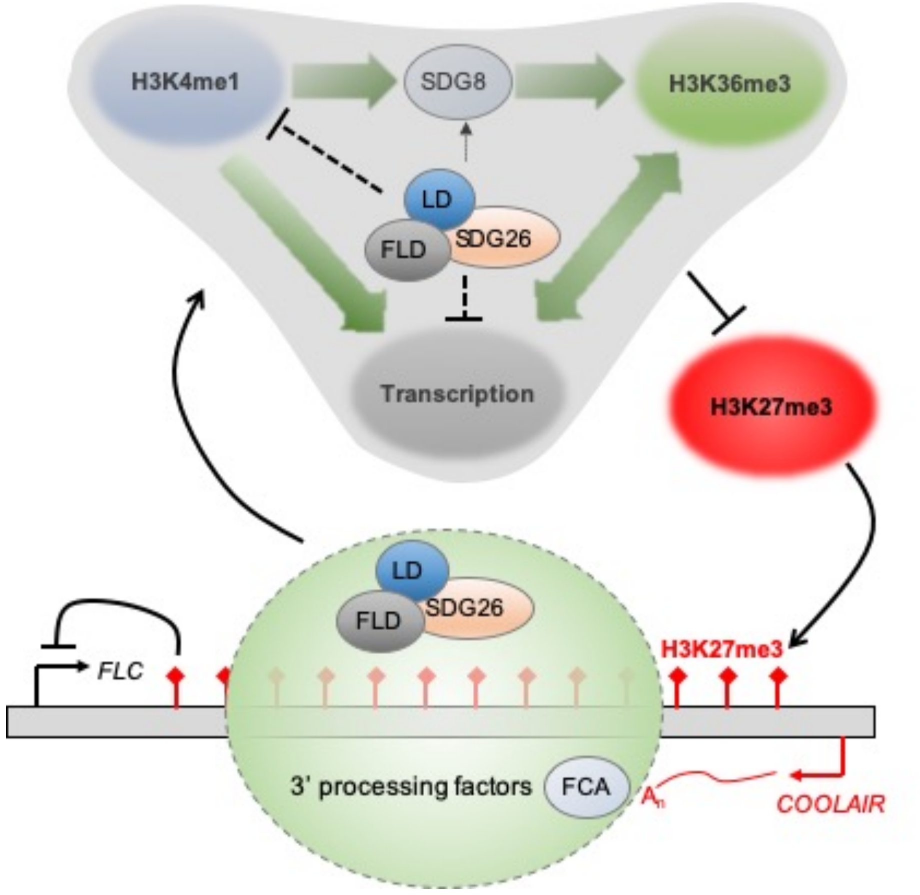

Fig. S5. A model depicting how RNA processing leads to chromatin silencing of *FLC* locus. When *COOLAIR* transcription occurs, the 3' processing mediated by FCA and 3' processing factors bring in the FLD/LD/SDG26-containing complex through their dynamic and transient interactions, potentially involving the liquid-like nuclear condensates. The FLD/LD/SDG26-containing complex inhibits an active transcription module involving H3K4me1, H3K36me3 and RNA Pol II. This module antagonizes the deposition of H3K27me3 such that its inhibition by the FLD/LD/SDG26-containing complex facilitates *FLC* silencing by H3K27me3.

Table S1. Primers used in this study

Primer name	Sequence (5' - 3')	Purpose
spFLC_F	AGCCAAGAAGACCGAACTCA	Amplification of spliced *FLC*
spFLC_R	TTTGTCCAGCAGGTGACATC	
unspFLC_F	CGCAATTTTCATAGCCCTTG	Amplification of unspliced *FLC*
unspFLC_R	CTTTGTAATCAAAGGTGGAGAGC	
UBC_F	CTGCGACTCAGGGAATCTTCTAA	Amplification of spliced *UBC*
UBC_R	TTGTGCCATTGAATTGAACCC	
FLC-a-F	actatgtaggcacgactttggtaac	ChIP-qPCR
FLC-a-R	tgcagaaagaacctccactctac	
FLC-b-F	GCCCGACGAAGAAAAAGTAG	
FLC-b-R	TTCAAGTCGCCGGAGATACT	
FLC-c-F	TGAAGTTTCAAGCCATCTTTGA	
FLC-c-R	TCACTCTGAAAAGAGACATTAATCA	
FLC-d-F	TTGACAATCCACAACCTCAATC	
FLC-d-R	TCAATTTCCTAGAGGCACCAA	
FLC-e-F	AGTTTGGCTTCCTCATACTTATGG	
FLC-e-R	CAATGAACCTTGAGGACAAGG	
FLC-f-F	ggggctgcgtttacatttta	
FLC-f-R	gtgatagcgctggctttgat	
FLC-g-F	TGAAATGTTACGAATACTAGCGTGT	
FLC-g-R	GGATCAAAACTACTAGCTAACCCTTG	
FLC-h-F	ccggttgttggacataactagg	
FLC-h-R	ccaaacccagacttaaccagac	
FLC-i-F	CCTGCTGGACAAATCTCCGA	
FLC-i-R	GGATTTTGATTTCAACCGCCGA	
FLC-j-F	AAGGTACAAAGTTCATCAACC	
FLC-j-R	cgtgtgagaattgcatcgag	

Dataset S1. List of proteins identified by FLD-FLAG-TAP affinity purifications.

Dataset S2. List of proteins identified by SDG26-GFP affinity purifications.

Dataset S3. List of proteins identified by GFP-LD affinity purifications.

Dataset S4. List of proteins identified by SDG26-GFP affinity purifications after crosslinking.

Dataset S5. List of proteins identified by GFP-LD affinity purifications after crosslinking.

SI References

1. Z. Wu *et al.*, Quantitative regulation of *FLC* via coordinated transcriptional initiation and elongation. *Proc Natl Acad Sci U S A* **113**, 218-223 (2016).
2. I. R. Henderson, F. Liu, S. Drea, G. G. Simpson, C. Dean, An allelic series reveals essential roles for FY in plant development in addition to flowering-time control. *Development* **132**, 3597-3607 (2005).
3. X. Fang *et al.*, Arabidopsis FLL2 promotes liquid-liquid phase separation of polyadenylation complexes. *Nature* **569**, 265-269 (2019).
4. P. Voigt *et al.*, Asymmetrically modified nucleosomes. *Cell* **151**, 181-193 (2012).
